
JOURNAL INFORMATION
==============================
Journal ID: Wirtschaftsdienst

Wirtschaftsdienst

EISSN: 1613-978X

ARTICLE INFORMATION
==============================
PMCID: PMC8933154
DOI: 10.1007/s10273-022-3128-1
Article ID: 3128
Article version: 1
Subjects: Zeitgespräch

Ursachen der Verbraucherverschuldung

Causes of Consumer Over-Indebtedness

Korczak Dieter dieter.korczak@gp-f.com

grid.418397.0 GP Forschungsgruppe, Breitscheidstr. 16, 16321 Bernau bei Berlin, Deutschland
Electronic publication date: 2022 Mar 19
Print publication date: 2022
Volume: 102
Issue: 3
First page: 170
Last page: 174
Copyright: © Der/die Autor:in 2022
License: Open Access: Dieser Artikel wird unter der Creative Commons Namensnennung 4.0 International Lizenz veröffentlicht (https://creativecommons.org/licenses/by/4.0/deed.de).
Open Access wird durch die ZBW — Leibniz-Informationszentrum Wirtschaft gefördert.
License URL: https://creativecommons.org/licenses/by/4.0/

Keywords: D18, G51
issue-copyright-statement: © The Author(s) 2022

==============================

Heiner Geißler (ehemaliger Bundesminister für Jugend, Familie und Gesundheit, Generalsekretär der CDU) hat bereits während seiner Zeit als Sozialminister in Rheinland-Pfalz 1967 bis 1977 den Begriff „neue soziale Frage" geprägt. Für ihn war diese neue Frage begründet in der strukturellen Armut im Sozialstaat. Seit 1974 wurde das wirtschafts- und sozialpolitische Ziel der Vollbeschäftigung nicht mehr erreicht. Die „neue" Armut war von daher im Wesentlichen durch Arbeitslosigkeit bedingt. Wohlfahrtsverbände hatten die strukturelle Armut mit ihren Angeboten zur Schuldnerberatung schon weitaus früher thematisiert. In Fürsorgeangebote integrierte Schuldnerberatungen sind seit 1958 zu beobachten. Die erste spezialisierte Schuldnerberatungsstelle nahm 1977 in Ludwigshafen (Rhein) ihre Arbeit auf.

Seitdem erfolgte ein kontinuierlicher Ausbau des Angebots auf derzeit rund 1.400 Insolvenz- und Schuldnerberatungsstellen. Im Zusammenhang mit Arbeitslosigkeit und einem Abwärtstrend bei den Einkommen der unteren Schichten entsteht zunehmend das Problem überschuldeter Haushalte (IFF, 1988, 22). Das umfassende allgemeingültige Kernelement von Überschuldung ist, dass finanzielle Forderungen aus eigener finanzieller Kraft, d.h. dem laufenden oder in naher Zukunft zu erwartenden Einkommen sowie aus der Auflösung von Sparguthaben und Vermögenswerten nicht (im vereinbarten Rahmen) getilgt werden können.

Überschuldung wird, je nachdem ob die psychologische, juristische, ökonomische oder soziologische Perspektive betont wird, unterschiedlich definiert. Eine weitere Differenzierung besteht zwischen administrativ-juristischen, objektiv-quantitativen und subjektiv-qualitativen Ansätzen. Die im europäischen Raum verwendeten unterschiedlichen Definitionen der Ver- und Überschuldung sind 2003 in einer Literaturrecherche verglichen worden. In einigen Definitionen wird das Unvermögen zur Erfüllung von Zahlungsverpflichtungen dahingehend konkretisiert, dass dies für den Fall gilt, wenn die Lebenshaltungskosten bereits in Abzug gebracht worden sind. Der krisenhafte soziale Prozess, der zu einer Überschuldung führt bzw. mit einer Überschuldung verbunden ist, wird von Korczak und Pfefferkorn (1992, XII) betont: „Überschuldung ist die Nichterfüllung von Zahlungsverpflichtungen, die zu einer ökonomischen und psychosozialen Destabilisierung von Schuldnern führt."

Sehr früh wurde auch über die Ursachen von Überschuldung nachgedacht. Groth (1984) hat als einer der ersten Schuldnerberater folgende Gründe und Ursachen zusammengetragen: zunehmende Arbeitslosigkeit, geringe frei verfügbare Einkommensanteile weiter Bevölkerungskreise, Zunahme des bargeldlosen Zahlungsverkehrs, monatliche Einkommensschwankungen, unvorhergesehene Ereignisse, Auswirkungen von reißerischen Werbeaussagen, sozialisationsbedingte Defizite im Bereich der privaten Haushaltsführung. Eine analytische Trennung dieser Gründe und Ursachen der Ver- bzw. Überschuldung erfolgte in dieser frühen Arbeit nicht. Ausgehend von der Theorie kritischer Lebensereignisse (Filipp, 1981) wurde von Reis (1988, 70) und Korczak und Pfefferkorn (1988; 1992, 274) zusätzlich zu Gründen und Ursachen der Ansatz der Überschuldungsauslöser (Trigger) eingeführt. Durch ein oder mehrere unvorhergesehene und nicht planbare Lebensereignisse entstehen Einkommensrückgänge und/oder finanzielle Mehrbelastungen, die bei einem eng bemessenen finanziellen Verfügungsspielraum in die Risikosituation einer Überschuldung führen. Oft werden durch das Lebensereignis Wendepunkte im Lebenslauf markiert. Je nachdem, welche Persönlichkeit in welcher biografischen Situation unter welchen situativen Bedingungen mit derartigen Zäsuren konfrontiert wird und wie sie darauf reagiert, sich auseinandersetzt, kann ein und dieselbe Grundsituation von manchen Individuen positiv, von anderen negativ erlebt werden und die weitere Entwicklung günstig oder ungünstig beeinflussen (Lehr, 1978, 163 f.).

Makro-soziologische und externe Faktoren, Kontextmerkmale, Coping-Strategien und Resilienz sind bei der Analyse von Überschuldungsursachen, Gründen und Auslösern ebenfalls zu berücksichtigen. Die Differenzierung in Ursachen, Gründe und Auslöser ist für die Analyse von Überschuldung und für die Beschreibung systematischer Zusammenhänge elementar.

## Ursachen der Überschuldung

Wenn zwei Phänomene ursächlich zusammenhängen, dann handelt es sich um einen Kausalzusammenhang. Die Ursache tritt zeitlich vor dem Ergebnis auf. Bei einer bestimmten

Ursache treten regelmäßig konkrete Folgen auf. Aufgrund des prozesshaften Verlaufs des Eintritts von Überschuldung und der Tatsache, dass in der Regel nicht einzelne Ursachen für Überschuldung ausschlaggebend sind, sondern ein Bündel von Faktoren, ist das Herausarbeiten von Gesetzmäßigkeiten und die Zuordnungen von Ursache und Wirkung bzw. Wenn-dann-Aussagen schwierig (Ansen, 2006; Korczak und Pfefferkorn, 1992; Zimmermann, 2000).

Die mittlerweile zahlreichen nationalen und europäischen Studien zur Entstehung von Überschuldung legen es nahe, von fünf Ursachen bzw. Ursachenfeldern auszugehen: Sozialisationsdefizite, prekäre Ressourcen, Allfinanzangebote, krisenhafte Lebensereignisse, Selbstständigkeit. Ursachen dienen der Erklärung, Gründe dem Verstehen von Handlungen (Davidson, 1985). Sie können einem Subjekt als Legitimation von Handlungen zugeschrieben werden. Gründe beeinflussen die Art und Weise der Reaktion. Bei der Berücksichtigung von Gründen wird das linear-kausale Interpretationsschema abgelöst vom Verständnis vernetzter Funktionalität in einer komplexen Umwelt.

## Auslöser der Überschuldung

Von Gründen streng zu trennen sind Auslöser der Überschuldung. Auslöser sind Ereignisse, die ein anderes Ereignis oder eine Ereigniskette in Gang setzen. Die Auslöser der Überschuldung, die 1992 erstmals systematisch identifiziert wurden, sind: Arbeitslosigkeit, Trennung/Scheidung, Probleme bei der Haushaltsführung, Niedrigeinkommen, Bildungsdefizite, Haushaltsgründung, Suchterkrankung, Unfall/Krankheit, Überversicherung, Nichtinanspruchnahme von Sozialdienstleistungen, Schwangerschaft. Als „persönlichkeitsbedingte" Faktoren wurden zusätzlich eine gewisse Naivität und Entwicklungs- und Erfahrungsdefizite genannt sowie kompensatorischer Konsum als Mittel zur Steigerung des Selbstwertgefühls, Unfähigkeit zu wirtschaftlicher Lebensplanung, Unerfahrenheit gegenüber Kredit und Konsumangebot sowie die Unfähigkeit, die eigenen Rechte durchzusetzen (Korczak und Pfefferkorn, 1992). Die vom Statistischen Bundesamt durchgeführten Erhebungen bei Schuldnerberatungsstellen nutzen die genannten Kategorien. Zu berücksichtigen ist bei dieser Statistik jedoch, dass nur der sogenannte Hauptauslöser der Überschuldung, der auf der Einschätzung der Schuldnerberater:innen beruht, aufgeführt ist (vgl. Tabelle 1).

Auch das Institut für Finanzdienstleistungen (iff) erstellt seit Jahren einen Überschuldungsreport, in dem es die Klientendaten von ausgewählten Schuldnerberatungsstellen analysiert (2020: 16.402 Beratungsfälle). Hier wird eine abweichende Struktur und Zuordnung der Termini verwendet. Anstelle von Überschuldungsanlässen spricht das iff von Überschuldungsgründen, die nach Ereignis, vermeidbarem Verhalten und anderen Ursachen aufgeschlüsselt werden. Als (kritisches) „Ereignis" gelten schwer vermeidbare externe Ereignisse wie Arbeitslosigkeit, Unfall, Krankheit, Scheidung/Trennung, Tod des Partners. Als „vermeidbares Verhalten" werden die Kategorien Konsumverhalten, unwirtschaftliche Haushaltsführung, fehlende finanzielle Allgemeinbildung, Straffälligkeit, Nichtinanspruchnahme von Sozialleistungen und unerlaubte Handlungen eingestuft. Als „andere Ursachen" werden ohne nähere Kategorisierung unter anderem Einkommensarmut, gescheiterte Selbstständigkeit, Haushaltsgründung etc. klassifiziert. Der Katalog der aufgezählten Überschuldungsauslöser beim iff ist identisch mit Destatis. Die Autoren schwanken in dem Überschuldungsreport jedoch zwischen den Bezeichnungen „Ursachen" und „Gründe" unsystematisch hin und her und verwischen dadurch wichtige Zusammenhänge.

Tabelle 1
**Hauptauslöser der Überschuldung/Schuldensumme von Schuldnerberatungskundschaft 2020**

| Hauptauslöser | Prozent-anteil | Schuldensumme pro Klient:in in Euro |
|---|---|---|
| Arbeitslosigkeit | 19,7 | 18.185 |
| Trennung/Scheidung/Tod des Partners | 12,0 | 34.547 |
| Erkrankung/Sucht/Unfall | 16,5 | 23.777 |
| Unwirtschaftliche Haushaltsführung | 14,5 | 21.656 |
| Längerfristiges Niedrigeinkommen | 9,6 | 17.097 |
| Gescheiterte Selbstständigkeit | 8,2 | 78.194 |
| Mithaftung etc. | 2,2 | 45.958 |
| Haushaltsgründung/Geburt | 2,1 | 17.950 |
| Gescheiterte Immobilienfinanzierung | 1,6 | 120.665 |
| Unerlaubte Handlungen | 1,4 | 39.887 |
| Unzureichende Kreditberatung | 1,3 | 37.020 |
| Nichtinanspruchnahme Sozialleistung | 0,7 | 14.260 |

Quelle: Destatis (2021).

## Ursache: Sozialisationsdefizite

Sozialisationsdefizite werden als Überschuldungsursache bei der Betrachtung von Biografien junger Überschuldeter offensichtlich, wie in einer qualitativen Pilotstudie gezeigt werden konnte. Die Jugendlichen und jungen Erwachsenen versuchen frühzeitig, einer belastenden Familiensituation zu entfliehen und einen positiven Gegenentwurf zu realisieren. Aufgrund mangelnder finanzieller, häufig auch sozialer und psychischer Ressourcen ist das Risiko des Scheiterns jedoch sehr hoch (vgl. Tabelle 2). Als Gründe für die Überschuldung von jungen Erwachsenen werden in den biografischen Interviews „Broken Home"-Konstellationen und/oder das Suchtverhalten der Eltern deutlich, das gepaart ist mit geringen finanziellen Mitteln. Die emotional und intellektuell vernachlässigte Betreuung der Kinder führt bei

Tabelle 2

**Fallbeispiele Überschuldungsbiografien**

| Männer | Frauen |
|---|---|
| Vater Alkoholiker – Eltern Scheidung – Realschulabschluss – mit 18 J. Auszug – Drogenkonsum – Abschluss mehrerer Handyverträge – Abbruch Lehrstellen – ALG II – mit 23 J. 6.000 Euro Schulden Inkasso, Handel, Handy, ÖPNV | Eltern geschieden – Mutter verschuldet – mit 17 J. zu Freund gezogen – Scham wegen Schulden – mit 18 J. Ehe + Kind – Ehemann ins Gefängnis – mit 20 J. Trennung – mit 21 J. 6.000 Euro Schulden Miete, Handel, GEZ, Energie |

Quelle: Korczak (2005a, 170 f.).

Tabelle 3

**Prekäre Einkommenssituationen als Ursache**

| Ursache | Niedrigeinkommen, prekäre Arbeitsverhältnisse |
|---|---|
| Gründe | Fehlender/niedriger Schulabschluss<br>geringe Entlohnung<br>befristete Arbeitsverhältnisse |
| Auslöser | Arbeitslosigkeit<br>Krankheit/Unfall<br>Kostenerhöhungen (z. B. Energie, Miete, Krankenkasse, Inflation)<br>Regulatorische Maßnahmen (siehe COVID-19-Maßnahmen) |

Quelle: eigene Zusammenstellung.

diesen zu unzureichender Finanzkompetenz und geringen finanziellen Skills. Bei den jungen Erwachsenen mit Sozialisationsdefiziten wird eine Überschuldung häufig durch eine verfrühte Haushaltsgründung ausgelöst, die vor dem Hintergrund von Niedrigeinkommen vorgenommen wird. Für die Führung des Haushalts benötigte Gegenstände werden häufig über den Versandhandel erworben, wobei das zu finanzierende Bestellvolumen zumeist die finanziellen Möglichkeiten übersteigt. Dies gilt auch für den Abschluss von überteuerten Handyverträgen. Sofern versucht wird, Mobilitätskosten durch Schwarzfahren einzusparen, werden zusätzliche Schulden kreiert. Peters (2019) weist darauf hin, dass junge Erwachsene ihre Probleme gern eigenverantwortlich lösen würden, aber überfordert sind.

## Ursache: Niedrigeinkommen/Prekarität

Schulz-Nieswand und Kurscheid (2007) sieht in einer prekären Einkommenslage einen chronischen Basisrisikofaktor. Reis (1988) bezeichnete diese Personengruppe als Armutsschuldner. Die Gründe für eine prekäre Einkommenssituation liegen darin, dass z. B. niedrige Bildungsabschlüsse zu schlechteren Einkommenschancen führen. Verstärkt ist zu beobachten, dass prekäre Arbeitsverhältnisse wie Zeitverträge, Teilzeit-Jobs, Mini-Jobs zunehmen und diese in der Regel nur mit dem gesetzlich vereinbarten Mindestlohn (2022: 9,82 Euro) vergütet werden. Das bedeutet einen Netto-Lohn von 1.283 Euro im Monat. Bei einer durchschnittlichen Mietbelastung von 30 % bis 40 % ist das Überschuldungsrisiko für einkommensschwache Personen und Haushalte eindeutig erkennbar. Auslöser für eine Überschuldung bei Niedrigeinkommensbeziehenden und Menschen in prekären Arbeitsverhältnissen ist nicht nur das Eintreten von Arbeitslosigkeit oder Krankheit, sondern es sind auch kaum steuerbare Kosten- oder Beitragserhöhungen z. B. bei Krankenkassenbeiträgen, Energiekosten, Mieterhöhungen und durch inflationäre Tendenzen.

Die regulatorischen Maßnahmen zur Bekämpfung der Coronapandemie haben gezeigt, dass auch durch staatliche Entscheidungen das private Überschuldungsrisiko erhöht werden kann. Eine finanzielle Überforderung vieler Menschen war bereits aufgrund von Kurzarbeit und Betriebsschließungen (Gastronomie, Hotel- und Beherbergungsgewerbe, Eventorganisationen, Kunst- und Kulturschaffende etc.) nach der ersten COVID-19-Welle nachweisbar. Insgesamt mussten 43 % der Befragten in den ersten sechs Monaten der Coronapandemie ihre finanziellen Ressourcen abbauen bzw. aufbrauchen (Korczak et al., 2021) (vgl. Tabelle 3).

## Ursache: Allfinanzangebot

33,7 % der Kundschaft von Schuldnerberatungsstellen haben Schulden aus Ratenkrediten und 28,8 % aus Dispo-Krediten. Durchschnittlich sind diese Klient:innen mit 23.132 Euro im Ratenkredit und 5.996 Euro im Dispo-Kredit verschuldet. Reifner hat darauf hingewiesen, dass die Nutzung geschäftsmäßiger Kreditformen Voraussetzung für die Teilnahme am gesellschaftlichen Leben und dem Zugang zu Auto, Haus und Liquidität in Krisenlagen ist. Prozedurale Armut tritt dann ein, „wenn der Gesamtkredit über den Gesamtsparmöglichkeiten in einem Leben liegt" (Reifner, 2003, 93). Die Ursache für Überschuldung liegt somit in der Nutzung der Finanzdienstleistungen. Wer keine Kredite aufnimmt, hat auch keine Schulden bei Finanzdienstleistern. Ein modernes Leben ohne die Nutzung von Finanzdienstleistern ist jedoch schwer bis gar nicht realisierbar. Gehälter werden auf Bankkonten überwiesen, Mieten und Versicherungsbeiträge von Bankkonten abgebucht, EC- und Kreditkarten ermöglichen den bargeldlosen Zahlungsverkehr.

Die Einführung des europäischen Binnenmarkts 1992 hat die Nutzung von Finanzdienstleistungen für den einzelnen Verbrauchenden jedoch verkompliziert. Unter dem Stichwort „Allfinanz" erfolgte ein Konzentrationsprozess bei Versicherungsunternehmen und Kreditinstituten, in dem diese zu einem Angebot gleicher oder ähnlicher integrierter Dienstleistungen mit finanziellen, geldwirtschaftlichen oder risikosichernden Funktionen übergegangen sind. Mit dem Auftreten von sogenannten FinTech-Unternehmen ist das Finanzdienstleistungsangebot noch komplexer geworden und hat die Möglichkeit, einen Überblick über diese Angebote und ihre Risiken zu erhalten, erschwert. Eine neue Stufe der integrierten komplexen Finanzangebote ist mit Embed-

Tabelle 4
### Allfinanzangebote als Ursache

| Ursachen | Inanspruchnahme von Finanzdienstleistungen |
|---|---|
| Gründe | Komplexes Finanzangebot/Aggressives Marketing<br>Embedded Finance/Buy now – Pay later<br>Tracking- und Tracing-Verfahren<br>Überschätzung des finanziellen Leistungsvermögens<br>Risikoanlagen, -kredite/schlechte Beratung |
| Auslöser | Einkommenseinbußen<br>Überblicksverlust<br>Spekulationsverluste |

Quelle: eigene Zusammenstellung.

ded Finance und „Buy now – Pay later“-Optionen erreicht worden. Bei Embedded Finance bekommen Nutzende alle (finanziellen) Dienstleistungen von einem Nicht-Banken-Anbieter. Für die Nutzenden von „Buy now – Pay later“ sind negative Konsequenzen bereits bekannt, 10 % von ihnen sind mit Inkasso-Unternehmen konfrontiert (Citizens Advice, 2021). Hinzu kommt der Verkauf risikoreicher Anlagen oder eine Kreditvergabe, die nicht in angemessener Weise die finanzielle Leistungsfähigkeit eines Kreditnehmenden berücksichtigt (Korczak, 2005b). Auslöser für das Eintreten einer Überschuldung sind dann der Verlust des Überblicks über die individuellen finanziellen Transaktionen, Eingehen riskanter Gewinnspekulationen, die scheitern oder Einkommenseinbußen bzw. -reduzierungen (vgl. Tabelle 4).

## Ursache: gescheiterte Selbstständigkeit

2020 gab es in Deutschland laut KfW 537.000 Existenzgründungen. Ein Drittel davon wird die Selbstständigkeit in den ersten drei Jahren wieder aufgeben, 27 % davon tun dies aus Unwirtschaftlichkeit einschließlich Insolvenz (KfW, 2021). Das Corona-Barometer der Steuerberatungsgesellschaft DATEV weist im Februar 2022 ein gravierendes Insolvenzrisiko für kleine und mittelständische Betriebe in Deutschland aufgrund der Auswirkungen der Pandemie aus. Hätte es die staatlichen Unterstützungsleistungen nicht gegeben, wären 22 % der Unternehmen von einer Insolvenz betroffen (DATEV, 2022). Diese Zahlen belegen das finanzielle Risiko, das mit einer Selbstständigkeit verbunden ist. Auch Freiberufler, Selbstständige oder Unternehmer können in die Überschuldung geraten. Seit 2012 wird für rund 8 % der Kundschaft von Schuldnerberatungsstellen eine gescheiterte Selbstständigkeit als Überschuldungsursache angegeben.

Die Gründe für das Scheitern einer Selbstständigkeit und das Eintreten von Überschuldung sind weitgehend identisch. Unzureichende kaufmännische Kenntnisse (Buchhaltung, Marketing) sind ein entscheidender Faktor. Häufig wird vergessen, Umsatzsteuern zu zahlen oder nicht berücksichtigt, dass Umsatzerlöse nicht gleich Einkommen sind. Oft fehlen bei der Gründung eines kleinen Unternehmens oder bei dem Gang in die Selbstständigkeit ausreichend finanzielle Mittel, um die Startphase mit geringen Erträgen finanziell überbrücken zu können. Selten wird auch vor Beginn der Selbstständigkeit eine Marktanalyse vorgenommen, die Standort, Produktpalette bzw. Leistungsangebot und Preise absichert. Externe Faktoren wie disruptive Technologien, denen sich der Leistungsanbieter bzw. Produzent nicht anpassen kann, sind ein weiterer Grund für das Scheitern einer Selbstständigkeit. Als Auslöser für den Eintritt einer Überschuldung wirken Umsatzrückgang oder Einkommenswegfall (z. B. durch Pandemiebekämpfungsmaßnahmen), der Einbruch von Lieferketten oder der Wegfall von Märkten, z. B. durch Sanktionen gegenüber bestimmten Ländern. Auch persönliche Faktoren wie das Eintreten eines Burnout aufgrund zu starker dauerhafter Belastung können zum Scheitern der Selbstständigkeit und dadurch zur Überschuldung führen (vgl. Tabelle 5).

Tabelle 5
### Gescheiterte Selbstständigkeit als Ursache

| Ursache | Gescheiterte Selbstständigkeit |
|---|---|
| Gründe | Fehleinschätzung des Marktes<br>unzureichende kaufmännische Kenntnisse<br>Finanzierungs-/Liquiditätsprobleme<br>Produktions-/Absatzprobleme<br>Disruptive Technologie |
| Auslöser | Umsatzrückgang/Einkommenswegfall<br>Einbruch Lieferketten<br>Wegfall von Märkten (z. B. Russland)<br>Gesetzgeberische Maßnahmen (z. B. COVID-19)<br>Burnout |

Quelle: eigene Zusammenstellung.

## Ursache: krisenhafte Lebensereignisse

Die wesentlichen Gründe für das Eintreten einer Krise sind Arbeitslosigkeit, Trennung, Scheidung oder Tod eines Partners bzw. Familienangehörigen. Außerdem akute oder chronische Erkrankungen und Unfälle. Die Krisen können durch das eigene Verhalten ausgelöst werden, können sich aber auch aus Struktur- und Rahmenbedingungen ergeben. Schulz-Nieswand und Kurscheid (2007, 66) hat den Eigenanteil als „habitualisiertes System der Daseinstechniken und Reaktionsstile in Bezug auf die exogenen An- und Herausforderungen im Lebenslauf“ formuliert. So kann z. B. Arbeitslosigkeit durch unzureichende Leistung oder fehlende Skills ausgelöst werden, aber auch durch Unternehmensübernahmen, Unternehmensumstrukturierungen oder wirtschaftspolitische Umorientierungen (z. B. Tagebauschließung in der Lausitz und am Niederrhein). Akute und chronische Krankheiten können durch gesundheitliches Fehlverhalten hervorgerufen werden, aber auch durch vom Individuum nicht beeinflussbare gesundheitsbelastende Arbeitsbedingungen und/oder Arbeitsverhältnisse. Münster et al. (2018) haben gezeigt, dass Krankheit zu Überschuldung ebenso wie Überschuldung zu Krankheit führen kann. Dass

Tabelle 6
**Krisenhafte Lebensereignisse als Ursache**

| Ursache | Krisenhafte Lebensereignisse |
|---|---|
| Gründe | Arbeitslosigkeit<br>Trennung/Scheidung/Tod des Partners, Familienangehöriger<br>Krankheit/Unfall |
| Auslöser | Unternehmensübernahme/-umstrukturierungen/-schließung<br>Fehlende Skills<br>Gesundheitliches Verhalten/Beeinträchtigungen<br>Dauer-Stress/Burnout<br>Auseinanderleben/Streit um Finanzen, Kindererziehung |

Quelle: eigene Zusammenstellung.

Dauerstress und Arbeitsverdichtung in einem Burnout resultieren können, ist ausreichend belegt (Korczak et al., 2012). Der Streit um die Verwendung der finanziellen Mittel stellt neben Auseinandersetzungen um Kindererziehung den häufigsten Trennungsgrund von (Ehe-)Paaren dar. Der Wegfall von Einkommen, die Kosten für getrennte Haushaltsführung, die Zahlung von Alimenten und Rechtsanwaltskosten sind in der Summe eine finanzielle Belastung, die insbesondere eine Scheidung zu einem der häufigsten Überschuldungsgründe macht (vgl. Tabelle 6).

## Abschließende Überlegung

Überschuldung ist im Kontext gesellschaftlicher Bedingungen, gesamtgesellschaftlicher Modernisierungsprozesse und gesellschaftspolitischer Umstände mit ihren wirtschaftlichen, rechtlichen und sozioökonomischen Auswirkungen einzuordnen. Diese Prozesse schaffen als sozialer Wandel neue Struktur- und Rahmenbedingungen, die wiederum neue Verarbeitungskompetenzen der Bevölkerung erfordern. Wesentlich für die Entwicklung von Überschuldung sind zum einen die wirtschaftliche und finanzielle Vulnerabilität der Menschen, Prekarität als Basisrisikofaktor für Überschuldung und zum anderen das Resilienzpotenzial von Personen.

Es kann gezeigt werden, dass es sich beim Eintreten von Überschuldung um einen zeitlichen Prozess handelt, der von dem subjektiven Gefühl der Überschuldung über den Zustand der relativen Überschuldung bis hin zur absoluten Überschuldung (Insolvenz) führt. Im Verlauf dieses Prozesses erfolgen Überlagerungen von sozialen, ökonomischen und individuellen Ursachen- und Auslöserkonstellationen (Schwarze, 2008, 218). „Over-indebtedness is both a cause and a consequence of poverty, deprivation and social exclusion... If over-indebtedness is not addressed in an appropriate and timely way, there can be negative consequences for affected individuals, households and society" (Eurofound, 2020, 1). Für eine angemessene soziologische Analyse der Überschuldung von Privatpersonen ist es daher zwingend notwendig, zwischen Ursachen, Gründen und Auslösern der Überschuldung zu unterscheiden.

Over-indebtedness of private households has been a problem in Germany for decades. Over-indebtedness depends on both the economic and financial vulnerability of people and their resilience potential. Social, economic and individual causes and triggers overlap in the process of over-indebtedness. Therefore, for the systematic analysis of over-indebtedness, a differentiation of the causes, reasons and triggers is essential. The article also presents examples.

Dr. Dieter Korczak ist Leiter der GP-Forschungsgruppe und Mitgründer des European Consumer Debt Network.


Literatur

Ansen, H. (2006), Soziale Beratung bei Armut, Ernst Reinhardt.
Citizens Advice (2021), Buy now — Pay later, https://www.citizensadvice.org.uk/about-us/about-us1/media/press-releases/one-in-10-buy-now-pay-later-shoppers-have-been-chased-by-debt-collectors/ (24. Februar 2022).
DATEV (2022), Corona-Barometer von Omikron geprägt, Pressemitteilung, 21. Februar.
Davidson, D. (1985), Handlungen, Gründe und Ursachen, Suhrkamp.
Destatis (2021), Statistik zur Überschuldung privater Personen 2020, Fachserie 15, Reihe 5, Statistisches Bundesamt.
Eurofound (2020), Adressing household over-indebtedness, EU Publication office.
Filipp, S.-H. (1981), Kritische Lebensereignisse, Urban&Schwarzenberg.
Groth, U. (1984), Schuldnerberatung. praktischer Leitfaden für die Sozialarbeit. Campus.
IFF — Institut für Finanzdienstleistungen und Verbraucherschutz (1988), Finanzdienstleistungen, soziale Diskriminierung und Verbraucherschutz, U. Reifner und W. Dörhage (Hrsg.), Mildner.
KfW Research (2021), KfW-Gründungsmonitor 2021.
Korczak, D. und G. Pfefferkorn (1988), Präsentation der Forschungskonzeption „Überschuldungsgutachten“ vor dem Forschungsbeirat des BMJ/BMFuS, GP-Forschungsgruppe.
Korczak, D. und G. Pfefferkorn (1992), Überschuldungssituation und Schuldnerberatung in der Bundesrepublik Deutschland, Schriftenreihe des Bundesministeriums für Familie und Senioren, Bd. 3, Kohlhammer.
Korczak, D. (2005a), Pilotstudie zur Überschuldung junger Erwachsener, in Schufa (Hrsg.), Schuldenkompass.
Korczak, D. (2005b), Verantwortungsvolle Kreditvergabe, Forschungsprojekt.
Korczak, D., M. Wastian und M. Schneider (2012), Therapie des Burnout Syndroms, Schriftenreihe Health Technology Assessment, Bd. 120, DIMDI.
Korczak D., S. Peters und H. Roggemann (2021), Private Überschuldung in Deutschland, WISO Diskurs, 07, Friedrich-Ebert-Stiftung.
Lehr, U. (1978), Das mittlere Erwachsenenalter — Ein vernachlässigtes Gebiet der Entwicklungspsychologie, in R. Oerter (Hrsg.) (1978), Entwicklung als lebenslanger Prozess, Hoffmann & Campe, 147–172.
Münster, E. et al. (2018), Arzneimittelkonsum, insbesondere Selbstmedikationbei überschuldeten Bürgerinnen und Bürgern in Nordrhein-Westfalen (ArSemÜ-Studie), Abschlussbericht Dezember 2018.
Peters, S. (2019), Armut und Überschuldung: Bewältigungshandeln von jungen Erwachsenen in finanziell schwierigen Situationen, Springer VS.
Peters, S. und H. Roggemann (2021), iff Überschuldungsreport 2021. Überschuldung in Deutschland, iff.
Reifner, U. (2003), Finanzielle Allgemeinbildung. Bildung als mittel der Armutsprävention in der Kreditgesellschaft, Nomos.
Reis C Verschuldung als Prozess. Kriminalsoziologische Bibliografie 1988 61 55 73
Schulz-Nieswand, F. und C. Kurscheid (2007), Die Schuld an der Schuld, Zur Überschuldung privater Haushalte, Merus.
Schwarze, U. (2008), Nachhaltige Sozialpolitik am Beispiel der Schuldnerberatung. Ziele, Qualitätsmerkmale und Vergleichsgrößen vor dem Hintergrund von Qualitätssicherung und Benchmarking, 1, NDV Mai, 214–219.
Zimmermann, G. E. (2000), Überschuldung privater Haushalte, Lambertus.
